# Clinicopathological characteristics and prognostic significance of casting-type calcifications in patients with invasive breast cancer presenting with microcalcification

**DOI:** 10.1038/s41598-024-64353-5

**Published:** 2024-06-10

**Authors:** Jiang Wang, Liangying Zhao, Xiaoshan Hu, Liting Lv, Xiaowei Zhang, Minjun Lu, Guinv Hu

**Affiliations:** 1 Department of Thyroid and Breast Surgery, Affiliated Dongyang Hospital of Wenzhou Medical University, Dongyang, 322100 Zhejiang China; 2 Department of Radiology, Affiliated Dongyang Hospital of Wenzhou Medical University, Dongyang, 322100 Zhejiang China; 3 Department of Pathology, Affiliated Dongyang Hospital of Wenzhou Medical University, Dongyang, 322100 Zhejiang China

**Keywords:** Anatomy, Biomarkers, Diseases, Medical research, Oncology, Risk factors

## Abstract

To explore the clinicopathological characteristics and prognostic significance of casting-type calcification (CC) in patients with breast cancer presenting with microcalcification on mammography. Data on patients with invasive breast cancer who had mammographic calcification was retrospectively analyzed. The chi-square test was utilized to assess the clinicopathological characteristics of two forms of CC-related breast cancer. The examination of prognostic variables was conducted using Kaplan–Meier and Cox regression analyses. A total of 427 eligible patients were included in this study. Chi-square analysis indicated that the presence of CC was associated with estrogen receptor (ER) negativity (P = 0.005), progesterone receptor (PR) negativity (P < 0.001), and epidermal growth factor receptor 2 (HER-2) positivity (P < 0.001); among these, the association was stronger with the CC-predominant type. After a median follow-up of 82 months, those with CC had a worse 5-year recurrence-free survival (RFS) (77.1% vs. 86.9%, p = 0.036; hazard ratio [HR], 1.86; 95% confidence interval [CI] 1.04–3.31) and overall survival (OS) (84.0% vs. 94.4%, p = 0.007; HR, 2.99; 95% CI 1.34–6.65) rates. In COX regression analysis, such differences were still observed in HER-2 positive subgroups (RFS: HR: 2.45, 95% CI 1–5.97, P = 0.049; OS: HR: 4.53, 95% CI 1.17–17.52, P = 0.029). In patients with invasive breast cancer exhibiting calcifications on mammography, the presence of CC, especially the CC-predominant type, is linked to a higher frequency of hormone receptor negativity and HER-2 positivity. The presence of CC is associated with an unfavorable 5-year RFS and OS rates.

## Introduction

Breast cancer is the most common malignant tumor among women worldwide, including in China, and one of the main causes of cancer-related deaths among women^1,2^. Mammography is the main imaging method for early breast cancer screening and the only imaging method that reduces breast cancer-related mortality^3^. Notably, 90% of patients with breast cancer detected through screening were positive on mammography, and studies have shown that 21% of patients with invasive breast cancer present with calcification on mammography^4^. Casting-type calcification (CC), shaped like a metal casting, is described as a linear or branching calcification in the Breast Imaging-Reporting and Data System (BI-RADS) and has a higher positive predictive value for breast cancer (approximately 70–100%) than other suspected calcifications. Furthermore, in the case of microcalcifications in a lesion with more than two morphological types, the BI-RADS category of the lesion was determined using the most suspicious morphological type^5,6^. Because of its distinguishing characteristics, CC has been studied frequently, and recent studies have suggested that breast cancer with CC is often identified as a type of breast cancer with neoductgenesis. Notably, the consistency in imaging and anatomy has been verified. Studies have shown that such type of breast cancer has invasive biological characteristics, is a more invasive cancer or high-grade intraductal carcinoma, and represents a special type of breast cancer^7,8^.

The mechanism underlying the formation of CC is complicated, and other types of calcifications may accompany it or co-occur with it. Only a few studies have explored the significance of CC-related breast cancer in a specific group of invasive breast cancers presenting with calcifications on mammography. Therefore, the present study aimed to retrospectively explore the clinicopathological features and prognostic significance of CC in patients with invasive breast cancer presenting with calcifications. We aimed to enable clinicians to obtain more information faster when making prognosis judgments and treatment decisions through the most easily obtained first-hand imaging data.

## Materials and methods

### Ethics statement

This study was approved by the independent ethics committee and institutional review board of Dongyang People’s Hospital. The requirement for written informed consent was waived by the committee owing to the study’s retrospective nature. We obtained permission from the Dongyang People’s Hospital to collect data from the Breast Surgery Department Database, under the approval protocol number 2021-YX-197. This study was conducted in compliance with the applicable guidelines and regulations.

### Patient selection

**Figure 1:**
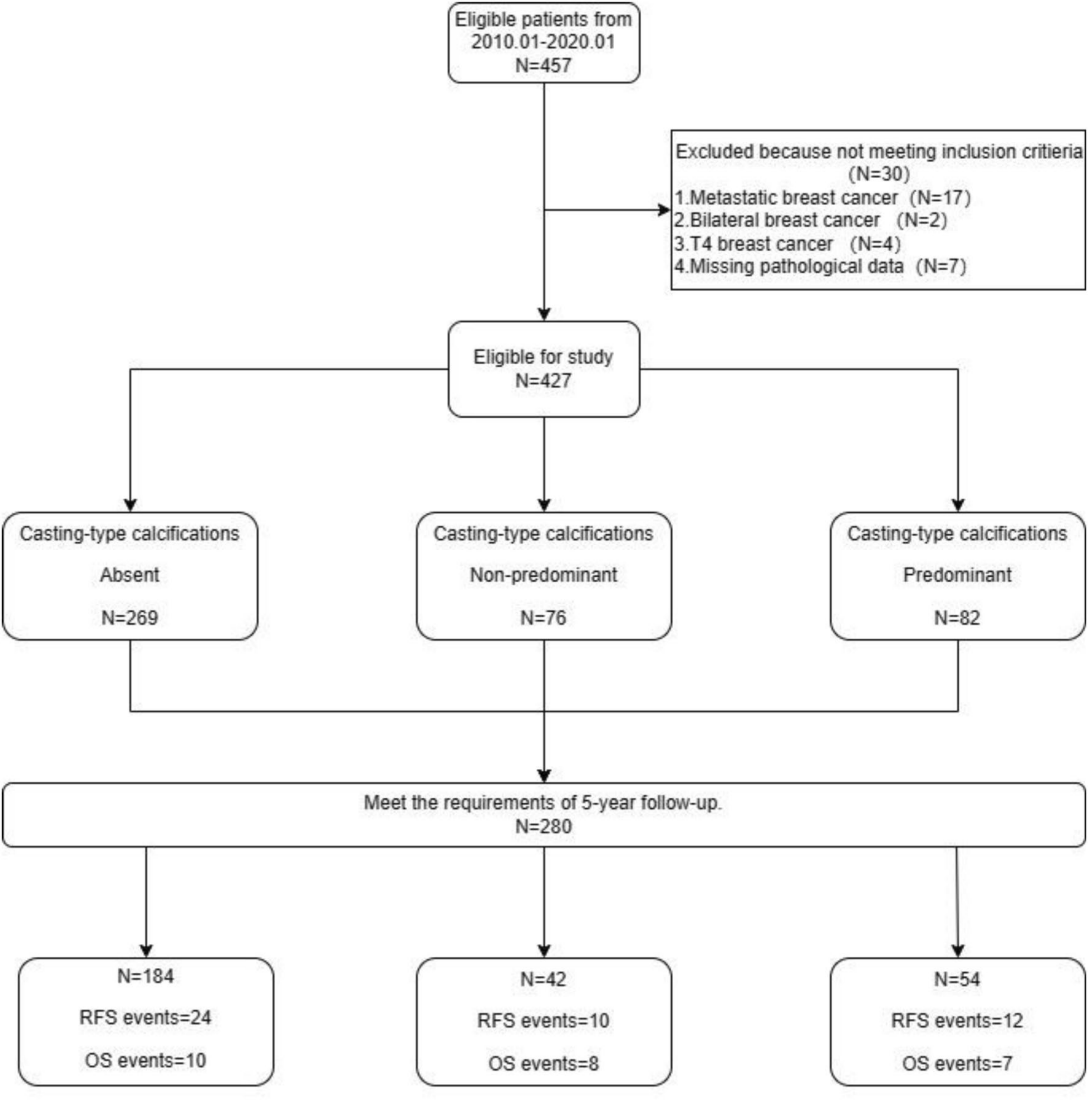
Diagram of the study population.

Between January 2010 and January 2020, 457 consecutive patients who underwent surgery at Dongyang People’s Hospital were enrolled in this study. These patients were carefully screened for subsequent analyses. The inclusion criteria were (1) history of surgery and pathological examination confirming the diagnosis of invasive breast cancer; (2) indication for mammography at the time of diagnosis, with a report describing suspicious calcification features, explained according to the BI-RADS classification^6^. The exclusion criteria were (1) diagnosis of breast cancer with distant metastasis; (2) diagnosis of bilateral breast cancer simultaneously or breast cancer at T4. Thirty ineligible patients were excluded (Fig. 1), and the remaining 427 patients were eligible for research on clinical characteristics.

This study explored the patients’ 5-year overall survival (OS) and recurrence-free survival (RFS) rates. OS was defined as the interval between treatment initiation and death. RFS was defined as the interval between treatment initiation and breast cancer recurrence or breast cancer-related death. Breast cancer recurrence involves both local and distant metastases. A total of 280 eligible patients had adequate medical information and were followed up (Fig. 1).

### Data collection

We retrospectively collected data on tumor characteristics and patients’ demographics from the medical records, including age, surgical methods, tumor grade, tumor size, and axillary lymph node, estrogen receptor (ER), progesterone receptor (PR), human epidermal growth factor receptor 2 (HER-2), and Ki67 index. Adjuvant or neoadjuvant therapy were administered following the guidelines of the National Comprehensive Cancer Network (NCCN) at that time. Treatments were selected based on patients' pathological markers and individual characteristics, including the use of anthracycline- or taxane-based regimens, endocrine therapy, and trastuzumab therapy. Pertuzumab has also been used to treat HER-2-positive breast cancer with lymph node involvement, considering the availability of the drug. Follow-up data from the center's follow-up system were also reviewed. Patients receiving treatment at the Dongyang People's Hospital were encouraged to make regular follow-up appointments. Patients who did not appear at the registered appointment were followed up through phone calls.

This study's analysis exclusively utilized pathological data derived from primary tumors, with neoadjuvant treatment data sourced from biopsies conducted prior to intervention. ER and PR were defined following the American Society of Clinical Oncology and College of American Pathologists Guidelines^9^, in which the positive threshold was indicated as ≥ 1%, and low ER expression was indicated as 1–10%. HER-2 status was tested using immunohistochemical staining (IHC) and was scored between 0 and + 3; tumors were considered negative if IHC scores were 0 or + 1 and positive if + 3. Tumors with + 2 scores were considered equivocal, requiring in situ hybridization^10^. Low expression of HER2 was defined as a score of 1+ on IHC analysis or as an IHC score of 2+ and negative results on in situ hybridization^11^.

Mammograms were acquired using a specialized digital mammography unit (MAMMOMAT Inspiration; Siemens Healthcare GmbH, Forchheim, Germany). Craniocaudal and mediolateral oblique mammograms were obtained for all patients. Additional or spot compression views were obtained as needed. Two experienced radiologists (HU and JIN) rechecked all images in cases of suspected calcification described in the report and was blinded to the patients’ survival status and other medical information.

### Definition of CC and method of grouping

**Figure 2:**
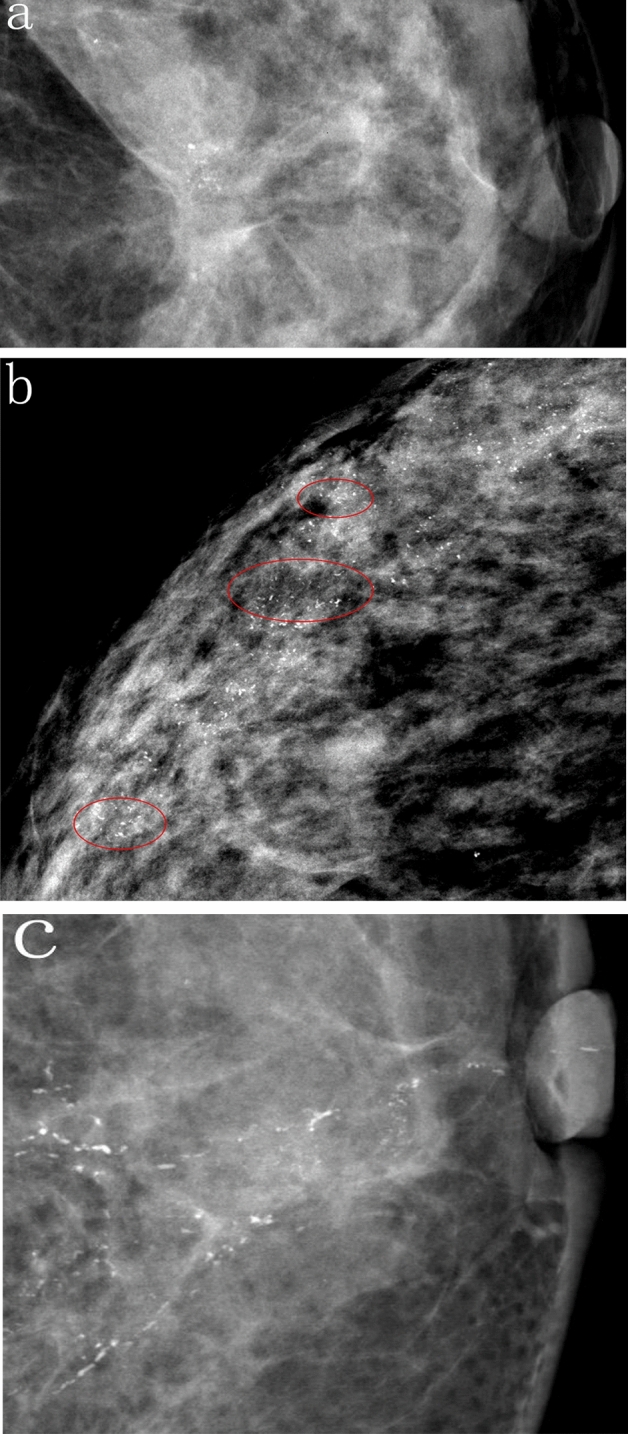
The three types of calcifications as defined in this study. (**a**) Left magnified mammogram of a 46-year-old woman. The craniocaudal images show clusters of predominantly amorphous microcalcifications. We classified this case as a CC-absent type. The final surgical pathology revealed HR-positive and HER-2-negative invasive breast cancer. (**b**) Right magnified mammogram of a 53-year-old woman. The craniocaudal images show predominant segmental pleomorphic and punctate microcalcifications, with CC scattered in them. We classified this case as a non-predominant type of CC. The final surgical pathology revealed HR-positive and HER-2-positive invasive breast cancers. (**c**) Left magnified mammogram of a 65-year-old woman. The craniocaudal images show predominantly regional classic CC. This case was classified as the predominant type of CC. The final surgical pathology revealed HR-negative and HER-2-positive invasive breast cancer.

As per the BI-RADS definition, CC are characterized by their thin, linear, and irregular shape, typically discontinuous and having a caliber smaller than 0.5 mm. In some cases, branching patterns were also observed^6^. In our study, we classified patients into three groups based on the performance level of the CC: the CC-absent group, the non-predominant CC group, and the predominant CC group. The categorization methodology employed in our study was delineated as follows: (1) The CC-absent Group (Fig. 2a): This group included a spectrum of calcifications characterized as non-fine-linear forms. These encompassed amorphous, coarse heterogeneous, or fine pleomorphic microcalcifications, as defined by the BI-RADS criteria. (2) The non-predominant CC Group (Fig. 2b): In this group, CC were significantly present in both Craniocaudal and mediolateral oblique mammograms. However, CC accounted for less than three-fourths of the total extent of calcifications observed. (3) The predominant CC Group (Fig. 2c): In this category, CC were conspicuously present, occupying at least three-fourths of the entire calcification area. In cases of disagreement regarding patient classification between two radiologists, a third radiologist (MEI) was consulted to facilitate a consensus-driven decision.

### Statistical analysis

Quantitative data were analyzed using Student’s t-test. Statistical analysis was conducted using the Chi-square test for the evaluation of categorical variables. The association strength and the direction of the relationships were assessed via Cramer's V values and standardized residuals, respectively. Pairwise comparisons among groups were corrected for multiple testing by employing the Bonferroni adjustment method. The Kaplan–Meier method was used to estimate the 5-year RFS and OS rates. A multivariate Cox proportional hazard regression model was used for multivariate survival analysis, adjusting for known prognostic factors affecting patients' survival, including age, histological grade, tumor size, lymph node status, ER, PR, and HER-2 expressions, and the presence of mass lesions on mammography. All statistical analyses were performed using SPSS Statistics (version 26.0; IBM Corp., Armonk, NY, USA), and statistical significance was set at p < 0.05.

## Results

### Clinicopathological characteristics of patients and CC-related breast cancer

This study included 427 eligible female patients diagnosed with invasive breast cancer and underwent surgical treatment at Dongyang People's Hospital. The age distribution for CC-absent group (median: 50 years, IQR: 13 years, range: 29–86 years) and CC-present group (median: 53 years, IQR: 16 years, range: 27–85 years) was assessed and found to be non-normally distributed. Table 1 presented participants’ demographical, clinical, and pathological characteristics. Of the 427 individuals who exhibited calcification features on mammography, 158 (37%) presented with CC (present group), whereas 269 (63%) did not (absent group). The age difference between the two groups was not statistically significant (P = 0.091). The Chi-square test revealed no significant differences in tumor size (P = 0.57), and axillary lymph node status (P = 0.138) between the two groups. Compared to the CC-absent group, the CC-present group had significantly higher rates of ER-negativity (38.6% vs. 25.7%, P = 0.005), PR-negativity (56.3% vs. 34.2%, P < 0.001), and HER-2 positivity (55.7% vs. 29.7%, P < 0.001), as well as a higher proportion of high-grade nuclear grading (P = 0.047). In comparing the CC-present group to the CC-absent group, there were fewer luminal A (7.6%) and Luminal B (HER2−) (16.5%) types, and more HER2 overexpressed (29.1%) types and Luminal-B (HER2+) (26.2%) types. In the field of radiology, the proportion of clustered distribution of CC present group is lower in the CC absent group (45.6% versus 72.9%), and the proportion of breast masses displayed in mammographic images was lower as well (63.9% versus 73.6%). In terms of surgical choice, the Mastectomy rate with CC-present group was significantly higher than that of the CC-absent group (81% vs 72.5%, P = 0.048). To further explore the existence of CC and its relationship with various clinicopathological indicators including the strength of association, this study calculated standardized residual values and Cramer's V statistic. The results are shown in Table 1, suggesting a moderate strength of association between CC and the expression of HER-2, molecular typing, and the distribution of calcification (Cramer's V > 0.3). The absolute values of standardized residuals in several cells exceeded 2, supporting the hypothesis of an association between CC and the corresponding clinicopathological indicators.

**Table 1:** Patient characteristics within subgroups.

Characteristic		Casting-type calcifications		P-value (χ^2^)	Cramer's V
		absent(n = 269)	present (n = 158)		
		(N, residual)	(N, residual)		
Median age (years)		51 ± 10	53 ± 11	0.091	–
Tumor grade	G1	33, 1.5	12, − 1.5	0.047	0.12
	G2	162, 1.2	86, − 1.2		
	G3	74, − 2.3	60, 2.3		
Tumor size	T1	127, 1	67, − 1	0.57	–
	T2	126, − 0.6	79, 0.6		
	T3	16, − 0.7	12, 0.7		
Axillary node metastasis	N0	150, 1.9	73, − 1.9	0.138	–
	N1	69, − 0.8	46, 0.8		
	N2+	50, − 1.5	39, 1.5		
ER	Negative	69, − 2.8	61, 2.8	0.005	0.136
	Positive	200, 2.8	97, − 2.8		
PR	Negative	92, − 4.5	89, 4.5	< 0.001	0.216
	Positive	177, 4.5	69, − 4.5		
HER2	Negative	174, 6.5	51, − 6.5	< 0.001	0.314
	Positive	80, − 5.3	88, 5.3		
	Indeterminate	15, − 2.4	19, 2.4		
Molecular typing	Luminal-A	72, 4.8	12, − 4.8	< 0.001	0.331
	Luminal-B (HER2−)	77, 2.8	26, − 2.8		
	Luminal-B (HER2+)	42, − 2.8	42, 2.8		
	HER2+	39, − 3.7	46, 3.7		
	TNBC	24, 0.2	13, − 0.2		
	Unknown	15, − 2.4	19, 2.4		
Surgery type	Mastectomy	195, − 2	128, 2	0.048	0.096
	BCS	74, 2	30, − 2		
Mass	Presence	198, 2.1	101, − 2.1	0.035	0.102
	Absence	71, − 2.1	57, 2.1		
Distribution	Regional	69, − 2.9	62, 2.9	< 0.001	0.332
	Grouped	196, 5.6	72, − 5.6		
	Linear	0, − 3.2	6, 3.2		
	Segmental	4, − 4.5	18, 4.5		

P < 0.05 indicates a significant difference. Residual Value: The difference between observed and expected frequencies. Cramer's V: Measures the strength of association between nominal variables, ranging from 0 (no association) to 1 (perfect association).

### Correlations between the three calcification types and clinicopathological features

**Figure 3:**
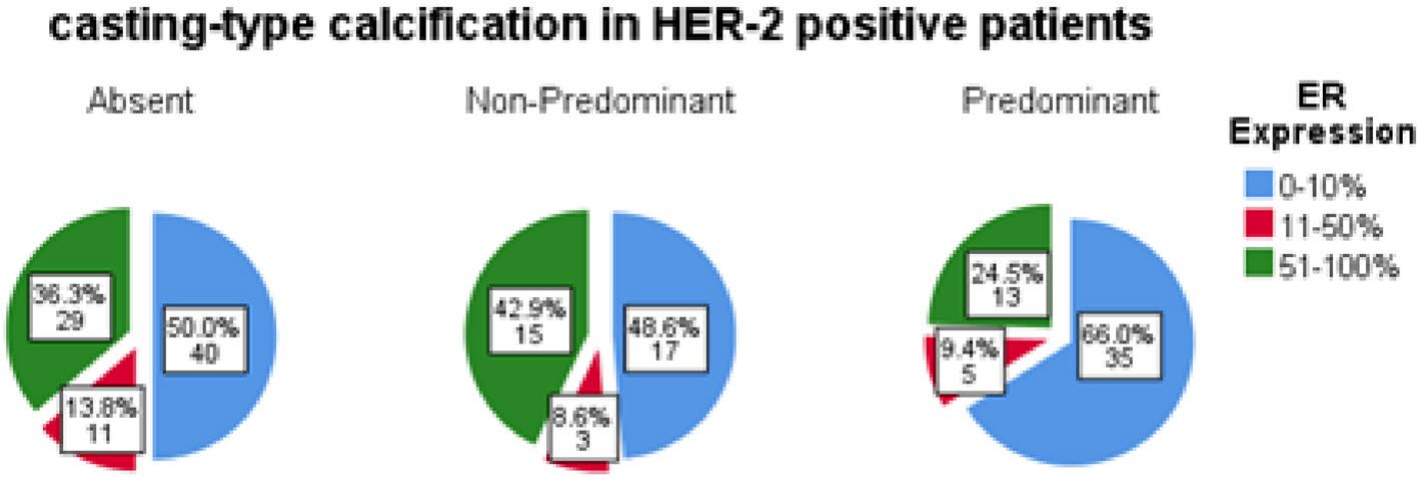
ER expression among three groups of HER-2 positive breast cancer.

According to the imaging manifestations of calcification, individuals were categorized into three groups: CC-absent group, CC-non-predominant group, and CC-predominant group. A total of six cases caused disagreements among radiologists regarding the classification based on the CC manifestations. Subsequently, a final decision was reached through in-depth discussions (Supplementary Fig. S1). Upon conducting an analysis of the clinicopathological characteristics among three patient cohorts, it was observed that there were no statistically significant differences with respect to tumor nuclear grading, tumor size, lymph node metastasis, and AJCC staging. In contrast, significant associations were identified in the expressions of ER (p = 0.002), PR (p < 0.001), HER-2 (p < 0.001), and Ki67 proliferation index (p < 0.001) across the groups, with Cramer's V values of 0.169, 0.278, 0.338, and 0.24, respectively, suggesting notable degrees of correlation. Pairwise intergroup comparisons were conducted among three patient cohorts utilizing the Bonferroni correction method, considering p-values < 0.0167 as indicative of significant differences. The findings indicated that, compared to the CC-absent group, the CC non-predominant group exhibited a significantly higher positive expression rate of HER-2 (p = 0.002) and a greater proportion of patients with a Ki67 proliferation index > 30% (p = 0.005). Similarly, relative to the CC-absent group, the CC-predominant group displayed significantly increased rates of ER negativity (p < 0.001), PR negativity (p < 0.001), HER-2 positivity (p < 0.001), and a higher proportion of elevated expression of the Ki67 index (p = 0.001). Additionally, when compared to the CC non-predominant group, the CC-predominant group demonstrated notably higher rates of PR negativity (p < 0.001) and HER-2 positivity (p = 0.005). It was noteworthy that, through pairwise comparisons among the three groups, the HER-2 positivity rate exhibited an incremental trend (Supplementary Table S1). A total of 168 cases of HER-2 positive breast cancer were included in the study, among which 92 patients had negative or low expression of ER. The CC-predominant group consisted of 35 individuals (66%), while the CC-non-predominant group consisted of 17 individuals (48.6%), and the CC-absent group consisted of 40 individuals (50%) (Fig. 3). The proportion of ER-negative or low expression in the CC-predominant group was significantly higher compared to the other two groups (66% vs. 49.6%, P = 0.046).

### Prognosis analysis

Among the 427 enrolled patients, 280 met the criteria for follow-up, with a median follow-up duration of 82 months (interquartile range: 66–106 months). This study primarily focused on evaluating the 5-year RFS) and OS rates. Specifically, the investigation encompassed 46 instances of breast cancer recurrence or breast cancer-related mortality (24 cases in the CC-absent group, 10 cases in the CC-non-predominant group, and 12 cases in the CC-predominant group). Furthermore, the cumulative 5-year mortality rate was observed in 25 patients (10 cases in the CC-absent group, 8 cases in the CC-non-predominant group, and 7 cases in the CC-predominant group) (Fig. 1).

**Figure 4:**
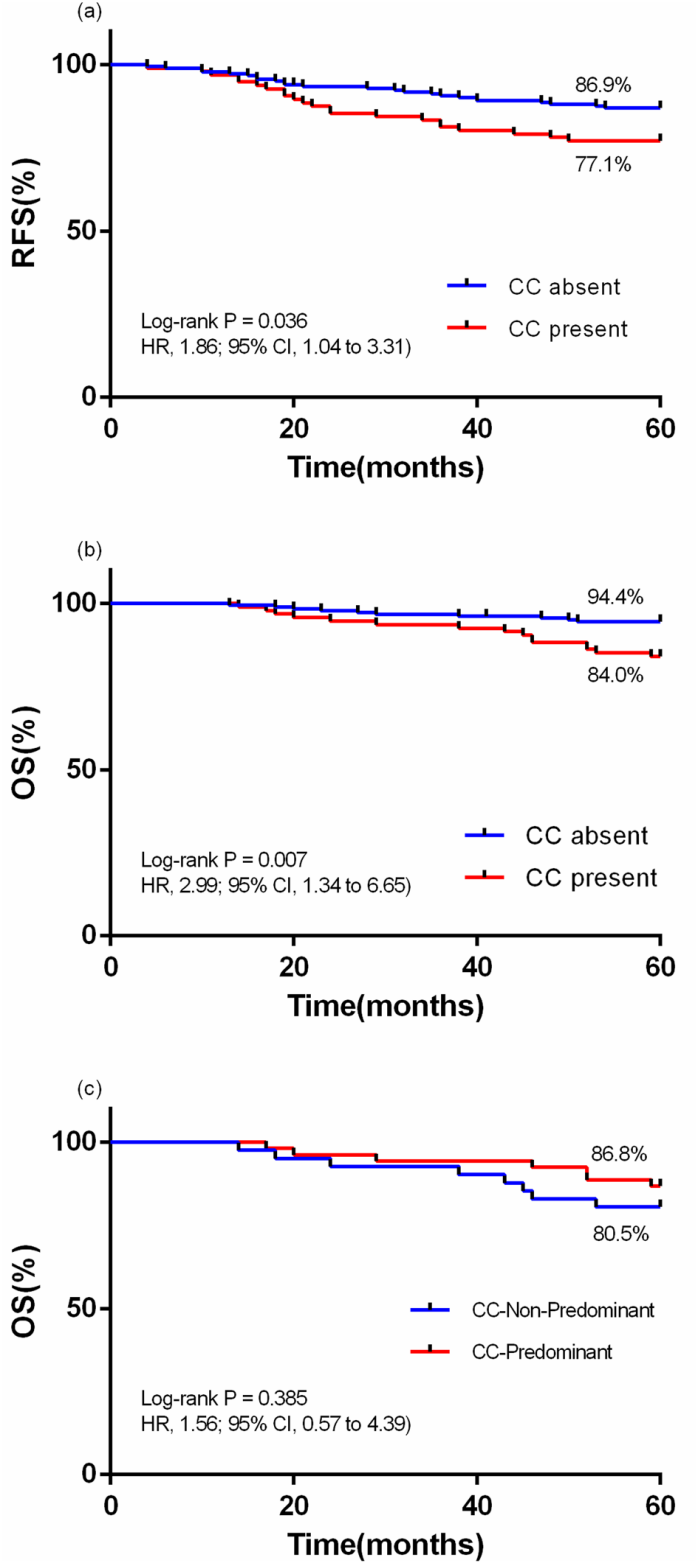
The influence of CC characteristics on the five-year prognostic outcome. (**a**) The 5-year RFS rates of patients with CC and those without CC (77.1% vs. 86.9%, p = 0.036); (**b**) The 5-year OS rates of patients with CC and those without CC (84.0% vs. 94.4%, p = 0.007). (**c**) The 5-year OS rates of CC-Non-Predominant group and CC-Predominant group were 80.5% and 86.8%, respectively (p = 0.385).

Notably, the CC-present group exhibited a significantly lower 5-year RFS rate compared to the CC-absent group (77.1% vs. 86.9%, p = 0.036; hazard ratio [HR], 1.86; 95% confidence interval [CI], 1.04–3.31) (Fig. 4a). Furthermore, the CC-present group demonstrated a notably lower 5-year OS rate compared to the CC-absent group (84.0% vs. 94.4%, P = 0.007; 95% CI 1.34–6.65) (Fig. 4b). The 5-year OS rates for the CC-non-predominant group and the CC-predominant were 80.5% and 86.8%, respectively, with no statistically significant difference observed (P = 0.385) (Fig. 4c).

The subgroup analysis reveals that significant prognostic differences associated with casting-type calcifications are predominantly concentrated within the cohort characterized by grade II, ER-positive, PR-positive, and HER2-positive status (Supplementary Table S3). To assess the proportional hazards assumption in our study, log–log survival plots were reviewed for each nominal covariate. The curves were examined for a roughly parallel orientation, suggesting that the proportional hazards assumption was generally met. Univariate survival analysis revealed that tumor nuclear grade, tumor size, lymph node, ER, and HER-2 expressions, and the presence of CC significantly impacted prognosis (Supplementary Table S2a). Adjusted COX regression analysis reveals that tumor size and axillary lymph node metastasis are independent prognostic factors, while the presence of CC does not demonstrate independent prognostic significance. (RFS: HR = 1.30, 95% CI 0.71–2.40, P = 0.4; OS: HR = 2.32, 95% CI 0.96–5.57, P = 0.061) (Supplementary Table S2b,c). Upon further investigation, it was found that within the HER-2 positive subgroup, the presence of CC was correlated with an unfavorable prognosis (RFS: HR = 2.45, 95% CI 1–5.97, P = 0.049; OS: HR = 4.53, 95% CI 1.17–17.52, P = 0.029) after controlling for other variables. Conversely, no statistically significant prognostic distinctions were observed within the HER-2 negative subgroup (Table 2).

**Table 2:** Influence of casting-type calcification on RFS and OS in different HER-2 status.

HER-2 status	5-year RFS rate			5-year OS rate		
	HR	95% CI	P	HR*	95% CI	P
Positive (n = 112)*	2.45	1–5.97	0.049	4.53	1.17–17.52	0.029
Negative (n = 145)*	0.64	0.19–2.09	0.457	2.49	0.47–13.35	0.287
All (n = 280)*	1.3	0.71–2.4	0.4	2.32	0.91–5.57	0.061

*Adjusted for tumor grade, tumor size, axillary node status, ER.

## Discussion

Mammography, a simple and quick screening method for breast cancer, has been widely used worldwide, and calcification is considered a characteristic of breast cancer. Notably, many studies believe breast cancer with calcification, especially CC-related breast cancer, is associated with a higher recurrence rate, worse prognosis, and higher prevalence of HER-2 positivity than breast cancer without calcification^12–17^. Presently, the categorization of calcifications adheres to the standards set forth in the BI-RADS of the American Radiological Society, with the fifth edition being the most current. This edition delineates types of calcifications potentially indicative of malignancy into categories such as amorphous, coarse heterogeneous, fine pleomorphic, and fine linear or fine-linear branching. It is important to note that within this framework, “pleomorphic calcifications” are specifically defined to exclude those of a fine-linear nature^6^. Scholars had simplified the classification of malignant calcifications into two categories based on the anatomy of the tumor: ductal central type (CC) and terminal ductal lobular unit (TDLU) central type^18^. Recent studies had reported that 75% of breast cancers originate from the TDLU rather than the main lactiferous duct according to tumor imaging and pathology, and calcification was mainly characterized by a cluster of crushed stone- and powder-like structures on imaging (Fig. 2a), which was indicated as acinar adenocarcinoma of the breast^5,19^. Furthermore, researchers posit that breast cancers associated with CC formations represent a distinct subtype within the spectrum of breast malignancies.^20,21^. Tabar et al. further classified ductal-origin breast cancers, categorizing them into six distinct types based on radiographic features. Among these, fragmented CC is identified as the most characteristic form^20,22^. In accordance with the BI-RADS terminology, these phenomena are characterized as fine linear and branching calcifications. Specifically, they manifest as densely packed fragmented CC, as illustrated in Fig. 2c. Additionally, they can appear as a dispersed array of fine linear and branching-like calcifications, exemplified in Fig. 2b. Tumors originating from ducts are frequently diagnosed in conjunction with those originating from TDLU. Notably, the ductal components of these tumors often exhibit a higher degree of invasiveness and are more inclined to express HER-2 positivity^21^. Typical calcification patterns corresponding to two distinct origins of tumor components are identified as casting-type calcification and crushed stone or powder-like calcifications. In clinical practice, it was often observed that CC tended to occupy a majority or even the entire range of calcification. Therefore, in our study, we decided to use three-quarters of the area as the threshold for distinguishing between predominant and non-predominant types, particularly when such tumors with invasive components were present. This might have represented a subset of tumors exhibiting distinct biological behaviors that affected the prognosis.

Academic researchers have consistently pursued in-depth investigations into the clinical and pathological characteristics of CC. A significant contribution to this field was made by Tabar et al., who reported that patients suffering from CC exhibited markedly poorer long-term survival rates in cases of invasive breast cancer, particularly when the tumor had a maximum diameter of 1-14 mm. However, it is critical to acknowledge that the study by Tabar et al. does not provide detailed information regarding the immunohistochemical properties of the infiltrative foci or DCIS, which could be pivotal in understanding the full scope of CC's clinical implications^16^. In an earlier study, Zunzunegui et al. observed that invasive tumors associated with 1–14 mm CC displayed a higher incidence of HER-2 receptor positivity (60%) and a greater frequency of HR receptor negativity (50%)^23^. These observations had been consistently supported by numerous subsequent studies, underscoring a higher prevalence of HER-2 positivity in CC-related breast cancer compared to both non-calcified breast cancer and non-CC-related calcified breast cancer^24–27^. Furthermore, research conducted by S.U et al. had shed light on the molecular aspects of breast cancer, specifically focusing on the ERBB2 gene. Their study revealed a significant up-regulation of mRNA expression levels of the ERBB2 gene in breast cancers characterized by CC. This up-regulation was markedly higher when compared to breast cancers with non-casting-type calcifications and those devoid of any suspicious calcifications^28^. Significantly, the observations from the current research align with these findings. Our study identified a notably HR negative rate and an increased HER-2 positive rate in patients diagnosed with CC-related breast cancer. These differences in HR and HER-2 status between the two groups underscore the distinct molecular profiles and potentially divergent pathological mechanisms characterizing CC-related and non-CC-related calcified breast cancers. Furthermore, this investigation delved into the distinct radiological presentations of breast cancer that arise from two separate anatomical regions within the breast, specifically focusing on the variation in calcification patterns observed. CC, which are generally representative of tumors originating from the ductal system, frequently coexist with calcifications commonly associated with TDLU. For the purposes of this study, patients who exhibited CC characteristics were categorized into two groups: the CC-predominant group and the CC-non-predominant group. Our research compared the predominant and non-predominant types in CC-related invasive breast cancer, revealing potential disparities in specific biomarkers between them. Pairwise inter-group comparisons revealed a trend towards a higher rate of negative ER expression in the CC-predominant type compared to the non-dominant, and a significantly greater negativity rate for PR expression was noted. Furthermore, the analysis showed that the positive rate of HER-2 expression in the non-CC type was significantly lower than in the non-predominant CC type, with the latter being substantially lower than in the CC-predominant type, depicting an ascending pattern. This finding was noteworthy since HER-2 overexpressing tumors are known to exhibit increased proliferation, invasion, and lymph node metastasis. Within the subgroup of HER-2 positive breast cancer patients, those predominated by CC displayed the highest frequency of low or negative ER expression. Importantly, in HER-2 positive breast cancer cases, the differential expression of ER may indicate distinct biological behaviors and prognostic outcomes between the two identified tumor types^29^.

A considerable body of scholarly research has been dedicated to understanding the prognosis of breast cancer associated with CC. Zunzunegui et al. observed that breast cancers associated with casting-type calcifications (CC), particularly those with small invasive tumors accompanied by extensive ductal carcinoma in situ (DCIS), exhibited a heightened incidence of lymph node positivity and a greater likelihood of necessitating systemic therapy^23^. Tabar et al. conducted a study on 714 patients diagnosed with invasive breast cancer, where the tumor size was confined to a maximum of 14 mm. Their findings revealed that the 20-year survival rate for patients in the CC group was markedly lower (52%) compared to those in the non-CC group, which ranged between 86 to 100%^16^. Additionally, Peacock et al. undertook a study involving 50 cases of CC-related invasive breast cancer, each measuring less than 15 mm. By matching these cases with a control group based on tumor size and lymph node status, they discovered that the recurrence rate in the CC group was significantly higher compared to the matched non-CC group^30^. However, it is crucial to acknowledge that the aforementioned studies primarily focused on small tumors measuring less than 15 mm, and the sample sizes were relatively restricted. Moreover, these investigations did not encompass anti-HER-2 therapies. Our study encompassed a substantial proportion of patients with T2 or higher staging, and all patients with HER-2 positive status underwent anti-HER-2 therapy.

Numerous investigations into invasive breast cancer with larger tumors have consistently demonstrated that CC-associated breast cancers have a lower rate of Recurrence-Free Survival (RFS) or Overall Survival (OS) compared to breast cancers not associated with CC^13,15,24,25,31^. Importantly, several of these studies have employed multivariate analyses and determined that CC constitutes an independent variable significantly influencing a poorer prognosis^13,31^. However, it should be noted that these cases are relatively older, and the data regarding the application or even the mention of anti-HER-2 treatments is limited or unavailable. YanLi et al. conducted an analysis of 136 cases of CC-related invasive breast cancer, representing the most extensive study to date. The results indicated that CC was an independent prognostic factor, given that all HER-2 positive patients had received standard anti-HER-2 therapy^14^. However, it was worth noting that, unlike their study, our investigation did not include breast cancers without calcification manifestations as comparators in the assessment of CC-associated breast cancer. Numerous studies have shown that breast cancers with microcalcifications exhibited a higher rate of HER-2 positivity^26,32^. Additionally, calcified breast cancer demonstrated significantly lower 8-year Recurrence-Free Survival (RFS) and OS rates compared to non-calcified breast cancer^15^. Limited research has been undertaken to compare the prognostic implications of invasive breast cancer with CC to other forms of calcifications, especially considering the extensive implementation of anti-HER-2 therapies.

In this study, we explored a cohort of patients diagnosed with invasive breast cancer, specifically those exhibiting calcification on diagnostic imaging. This approach allowed for the exclusion of confounding factors typically associated with calcifications in breast cancer. Our investigation yielded significant insights into the subgroup of patients with CC-characterized breast cancer. These patients demonstrated higher rates of HR negativity and increased HER-2 positivity, indicating a propensity for enhanced HER-2 overexpression and a potentially more aggressive biological profile. In this study, all patients with HER-2 positive status underwent adjuvant therapy based on Trastuzumab; post-2016, patients with lymph node positivity additionally received Pertuzumab treatment. Analysis employing Kaplan–Meier survival curves demonstrates that, compared to other calcified breast cancers, breast cancers associated with CC exhibited lower 5-year rates for RFS and OS. Interestingly, the prognosis of breast cancers with a predominant CC presentation does not appear to be worse than that of tumors with a non-dominant CC manifestation. Notably, this prognostic difference was more pronounced in subgroups characterized by nuclear grade 2, hormone receptor positivity, and HER-2 positivity. However, when adjusting for a spectrum of other prognostic factors, CC was not conclusively identified as an independent prognostic factor in comparison to other types of breast calcifications. The subgroup analysis of the HER-2 positive cohort, which was controlled for various prognostic factors, revealed a noteworthy finding. Patients with CC exhibited a markedly worse prognosis compared to those without CC. However, such a discrepancy was not evident within the HER-2 negative subgroup.

In this scholarly examination, we investigated the hypothesis that CC in breast cancer is linked to increased tumor aggressiveness and metastatic potential. This phenomenon is thought to be associated with tumor stem cells involved in new ductal formation, which exhibit resistance to systemic therapies and radiotherapy. These observations prompt an inquiry into whether breast cancers exhibiting CC are particularly resistant to HER-2 targeted therapies^21,33,34^. Considering the ongoing development of anti-HER-2 medications, further research is imperative to accurately delineate the subset of breast cancer patients with CC, thereby optimizing therapeutic strategies. Casting-type Calcifications, particularly in their predominant form, represent a readily accessible pre-treatment imaging feature that can be accurately identified even by clinicians with limited experience. In addition to their evident radiographic features, breast cancers associated with CC, in contrast to those presenting other forms of calcifications, demonstrate worse biological behaviors and poorer prognostic outcomes, warranting further in-depth investigation.

This study has several limitations: a single-center cohort limiting event numbers, limited statistical power, and survival curve interpretations without reaching the median survival. A multicenter study with a larger sample is recommended to validate the findings related to Casting-type Calcifications in breast cancer.

## Conclusions

This investigation focused on the distinct biological traits and prognostic significance of CC-related breast cancer, particularly in subjects displaying mammographic calcifications. To accurately assess the specific effects of CC, the study purposefully excluded the broader influence of calcifications in general. It emerged that breast cancers with CC tend to exhibit a marked inclination towards HR negativity and HER-2 positivity. This trend was especially evident in cases predominantly marked by CC. In breast cancer with calcifications, breast cancer associated with Casting-type Calcifications tends to have poorer 5-year RFS and OS rates. After adjusting for other prognostic factors, exploratory findings still observed such disparities in the HER-2 positive subgroup. These findings highlight the imperative need for in-depth research into the distinct characteristics and clinical implications of CC-related breast cancer, focusing on its recognizable imaging features and aggressive nature.

### Supplementary Information

Supplementary Information.

## Data Availability

Upon a reasonable request, the primary data underpinning the conclusions of this manuscript will be provided by the corresponding author.
