# Supplementary material for: Clinicopathological characteristics and prognostic significance of casting-type calcifications in patients with invasive breast cancer presenting with microcalcification
Source: Sci Rep. 2024 Jun 10;14:13351. doi: 10.1038/s41598-024-64353-5 (PMC11164990; doi:10.1038/s41598-024-64353-5)
Supplement: Supplementary file 1 — Supplementary Information. [file 41598_2024_64353_MOESM1_ESM.pdf]

Supplementary Table S1:

Characteristic		Casting-type calcification			P-value (χ²)	Cramer's V
		Absent (N=269)	Non-Predominant (N=75)	Predominant (N=83)		
		(N, Residual)	(N, Residual)	(N, Residual)		
**Tumor grade**	G1	33,1.5	7,-0.4	5,-1.5	0.153	—
	G2	162,1.2	41,-0.7	45,-0.8		
	G3	74,-2.3	27,0.9	33,1.8		
**Tumor size**	T1	127,1	37,0.7	30,-1.9	0.216	—
	T2	126,-0.6	35,-0.3	44,1		
	T3	16,-0.7	3,-1	9,1.8		
**Axillary node metastasis**	N0	150,1.9	39,0	34,-2.3	0.13	—
	N1	69,-0.8	17,-0.9	29,1.8		
	N2+	50,-1.5	19,1.1	20,0.8		
**ER**	Negative	69,-2.8	23,0	38,3.4	0.002	0.169
	Positive	200,2.8	52,0	45,-3.4		
	$P^{\#}$	P^a=0.385	P^b=0.051	$P^c < 0.001^*$		
**PR**	Negative	92,-4.5	31,-0.2	58,5.6	< 0.001	0.278
	Positive	177,4.5	44,0.2	25,-5.6		
	$P^{\#}$	P^a=0.254	$P^b < 0.001^*$	$P^c < 0.001^*$		
**Ki67**	Ki67 < 20%	71,4	10,-2.1	10,-2.8	< 0.001	0.24
	Ki67 > 30%	99,-4	40,2.1	47,2.8		
	$P^{\#}$	P^a=0.005*	P^b=0.745	P^c=0.001*		
**HER2**	Negative	174,6.1	33,-1.6	18,-6	< 0.001	0.338
	Positive	80,-6.1	35,1.6	53,6.0		
	$P^{\#}$	P^a=0.002*	$P^{b=}$0.005*	$P^c < 0.001^*$		
**AJCC Stage**	Stage I	115,0.8	35,1.1	26,-2	0.201	—
	Stage II	105,0.3	24,-1.3	35,0.8		
	Stage III	49,-1.4	16.0.2	22,1.5		

"$P^{\#}$" represents P-values for pairwise comparisons: "P^a" for absent group vs. non-predominant group, "P^b" for non-predominant group vs. predominant group, "P^c" for absent group vs. predominant group. After Bonferroni correction for multiple comparisons, the significance level was adjusted to 0.0167. Groups marked with an asterisk (*) indicate pairwise comparisons where the original P-value reached this level of significance.

Considering that our patients had completed standardized anti-tumor therapy, we used the 8th edition of the AJCC Cancer Staging Manual as the tumor staging basis for all patients. In this study, patients with undetermined HER-2 status were not administered anti-HER-2 therapy. Discrepancies between the 8th edition AJCC staging and anatomical staging defer to the latter.

Supplementary Table S2a：

Univariate analysis	5-year RFS rate		5-year OS rate	
	HR (95%CI)	P	HR (95%CI)	P
**Age**				
**＜40**	Reference			
**40-65**	1.2(0.47-3.07)	0.701	3.51(0.47-26.12)	0.221
**＞65**	1.85(0.56-6.05)	0.311	5.98(0.67-53.46)	0.11
**Tumor grade**				
**G1-2**	Reference			
**G3**	2.68(1.50-4.78)	0.001	2.37(1.08-5.20)	0.031
**Tumor size**				
**T1**	Reference			
**T2**	2.41(1.19-4.90)	0.015	3.76(1.24-11.43)	0.019
**T3**	7.9(3.35-18.63)	< 0.001	14.04(4.10-48.03)	< 0.001
**Axillary node metastasis,**				
**N0**	Reference			
**N1**	1.43(0.58-3.58)	0.434	6.68(1.39-32.13)	0.018
**N2**	5.35(2.35-12.12)	< 0.001	11.07(2.15-57.04)	0.004
**N3**	12.63(5.78-27.60)	< 0.001	41.32(9.15-186.70)	< 0.001
**ER**				
**Positive**	Reference			
**Negative**	2.01(1.13-3.58)	0.018	2.32(1.06-5.09)	0.035
**PR**				
**Positive**	Reference			
**Negative**	1.63(0.91-2.92)	0.097	2.39(1.06-5.40)	0.037
**HER2**				
**Negative**	Reference			
**Positive**	2.18(1.19-3.99)	0.012	2.73(1.17-6.37)	0.021
**Indeterminate**	0.73(0.17-3.16)	0.673	0.78(0.10-6.27)	0.818
**Mass**				
**Absent**	Reference			
**Present**	1.30(0.64-2.61)	0.47	1.87(0.64-5.45)	0.251
**Casting-type calcification model1**				
**Absent**	Reference			
**Present**	1.86(1.04-3.31)	0.036	2.99(1.34-6.65)	0.007
**Casting-type calcification model2**				
**Absent**	Reference			
**Non-Predominant**	2.00(0.96-4.19)	0.065	3.76(1.48-9.53)	0.005
**Predominant**	1.75(0.88-3.50)	0.113	2.42(0.92-6.36)	0.073

Supplementary Table S2b：

Multivariate analysis	5-year RFS rate		5-year OS rate	
	HR (95%CI)	P	HR (95%CI)	P
**Tumor grade**				
**G1-2**	Reference			
**G3**	1.60(0.83-3.09)	0.161	1(0.40-2.51)	1
**Tumor size**				
**T1**	Reference			
**T2**	1.50(0.72-3.13)	0.281	2.08(0.67-6.44)	0.203
**T3**	4.98(1.98-12.54)	0.001	4.66(1.26-17.32)	0.021
**Axillary node metastasis,**				
**N0**	Reference			
**N1**	0.88(0.34-2.29)	0.796	3.68(0.72-18.74)	0.116
**N2**	3.21(1.35-7.64)	0.009	5.47(1.01-29.56)	0.048
**N3**	7.89(3.44-18.06)	< 0.001	25.92(5.27-127.44)	< 0.001
**ER**				
**Positive**	Reference			
**Negative**	1.63(0.86-3.06)	0.133	1.92(0.81-4.54)	0.136
**HER2**				
**Negative**	Reference			
**Positive**	1.16(0.56-2.40)	0.694	1.32(0.48-3.62)	0.593
**Indeterminate**	0.61(0.14-2.71)	0.519	0.79(0.10-6.59)	0.828
**Casting-type calcification model1**				
**Absent**	Reference			
**Present**	1.30(0.71-2.40)	0.400	2.32(0.96-5.57)	0.061

Supplementary Table S2c：

Multivariate analysis	5-year RFS rate		5-year OS rate	
	HR (95%CI)	P	HR (95%CI)	P
**Tumor grade**				
**G1-2**	Reference			
**G3**	1.55(0.80-3.00)	0.191	0.86(0.33-2.23)	0.756
**Tumor size**				
**T1**	Reference			
**T2**	1.53(0.73-3.21)	0.256	2.23(0.71-6.98)	0.168
**T3**	5.23(2.08-13.13)	< 0.001	4.81(1.32-17.57)	0.017
**Axillary node metastasis,**				
**N0**	Reference			
**N1**	0.90(0.35-2.34)	0.833	4.05(0.80-20.61)	0.092
**N2**	3.31(1.39-7.88)	0.007	5.77(1.07-31.09)	0.041
**N3**	8.04(3.49-18.53)	< 0.001	27.35(5.46-137.34)	< 0.001
**ER**				
**Positive**	Reference			
**Negative**	1.70(0.90-3.21)	0.103	2.15(0.90-5.11)	0.084
**HER2**				
**Negative**	Reference			
**Positive**	1.18(0.58-2.43)	0.645	1.42(0.53-3.77)	0.488
**Indeterminate**	0.67(0.15-2.96)	0.594	0.94(0.11-7.83)	0.954
**Casting-type calcification model2**				
**Absent**	Reference			
**Non-Predominant**	1.73(0.81-3.69)	0.159	3.69(1.33-10.21)	0.012
**Predominant**	1.06(0.51-2.20)	0.884	1.62(0.57-4.58)	0.366

Table S2: Single-factor COX analysis (**Table S2a**) and multi-factor COX analysis (**Table S2b、S2c**) of the 5-year RFS rate and OS rate. HR, hazard ratio.

Supplementary Table S3:

Subgroup		5-year RFS			5-year OS		
		Non-CC %	CC %	P-value	Non-CC %	CC %	P-value
**Tumor grade**	I	94.7	100	0.544	100	100	/
	II	91.5	76.5	**0.006**	98.3	78	**<0.001**
	III	71.4	73.7	0.769	81.8	89.2	0.344
**Tumor size**	T1	93.4	86.8	0.21	98.9	92.1	**0.043**
	T2	85.1	73.5	0.107	92.3	83	0.114
	T3	50	55.6	0.741	71.6	55.6	0.516
**Axillary node**	Negative	92.3	92.7	0.959	99	97.4	0.487
	Positive	79.9	65.5	0.067	88.6	74.5	**0.039**
**ER**	Negative	76.8	75	0.855	87	84.1	0.747
	Positive	90.4	78.8	**0.03**	97	84	**0.001**
**PR**	Negative	81.1	77.6	0.674	87.1	86.2	0.937
	Positive	90	76.3	**0.023**	98.3	80.6	**<0.001**
**HER2**	Negative	88.7	86.2	0.684	95.6	89.3	0.181
	Positive	84.2	67.3	**0.037**	92.6	77.8	**0.033**

Table S3: Subgroup analysis for 5-year outcome differences between patients with and without CC. The bold text indicates statistical significance (P<0.05).

Supplementary FigS1: Six images selected following deliberation on controversial categorization.

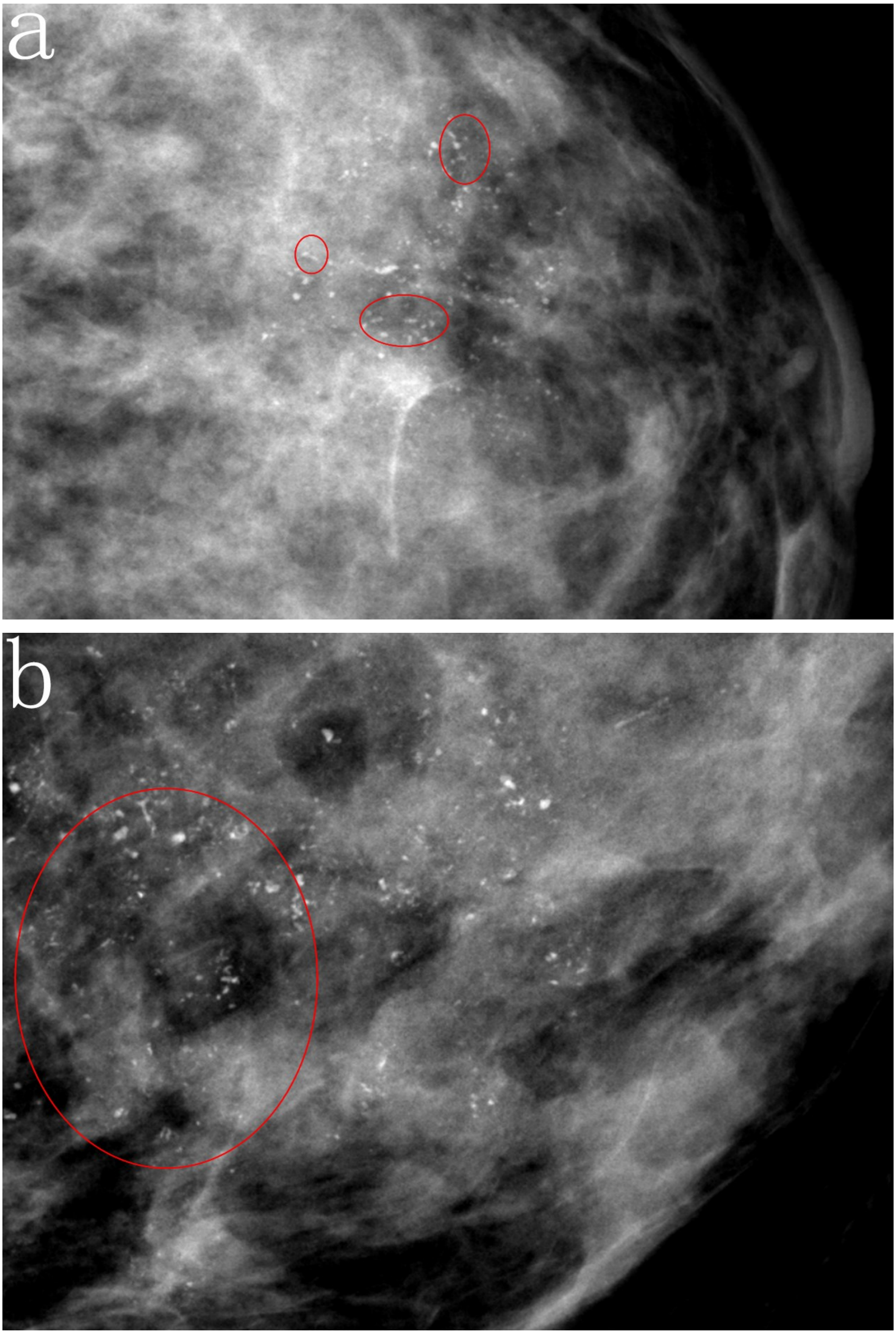

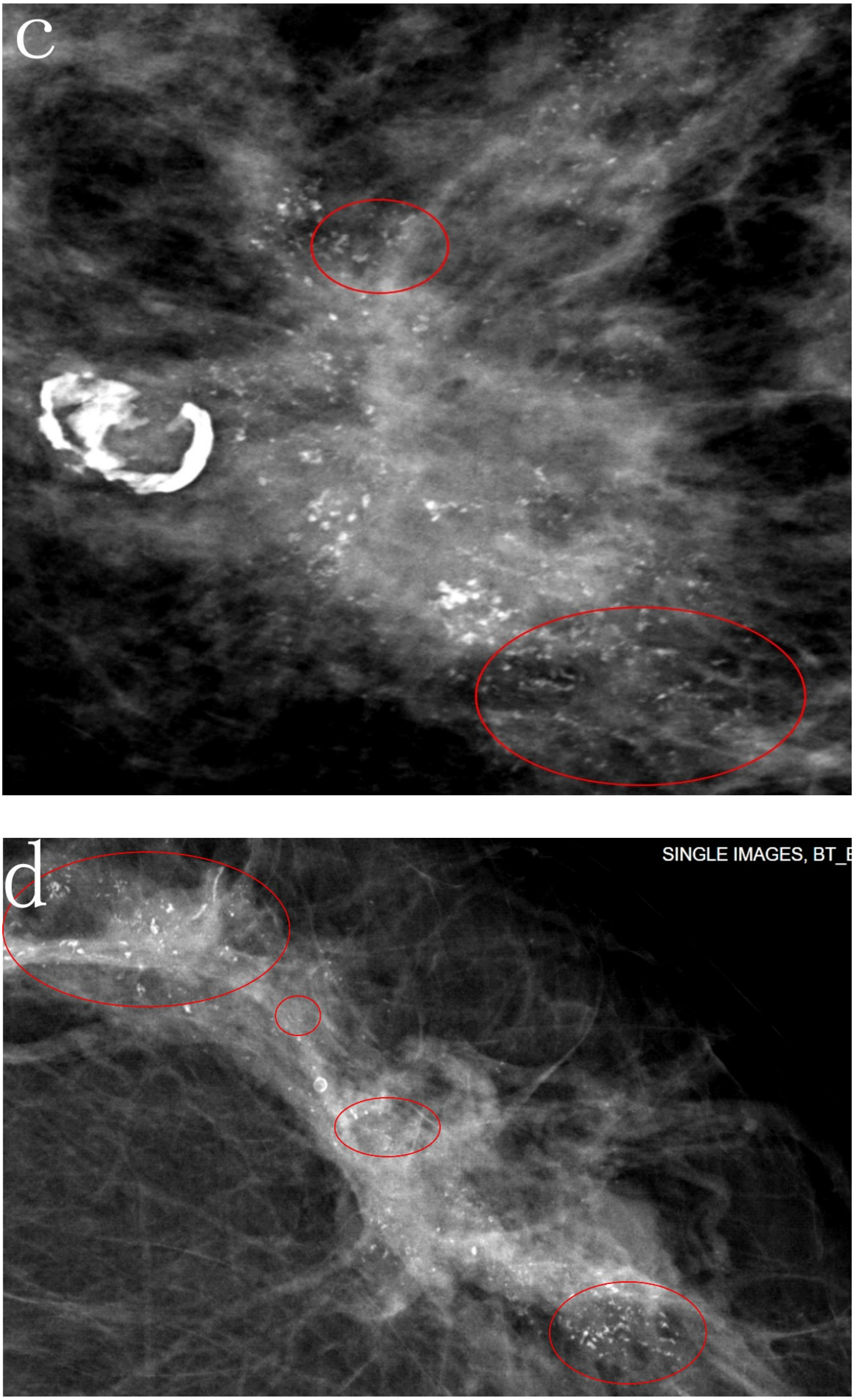
c
d
SINGLE IMAGES, BT_

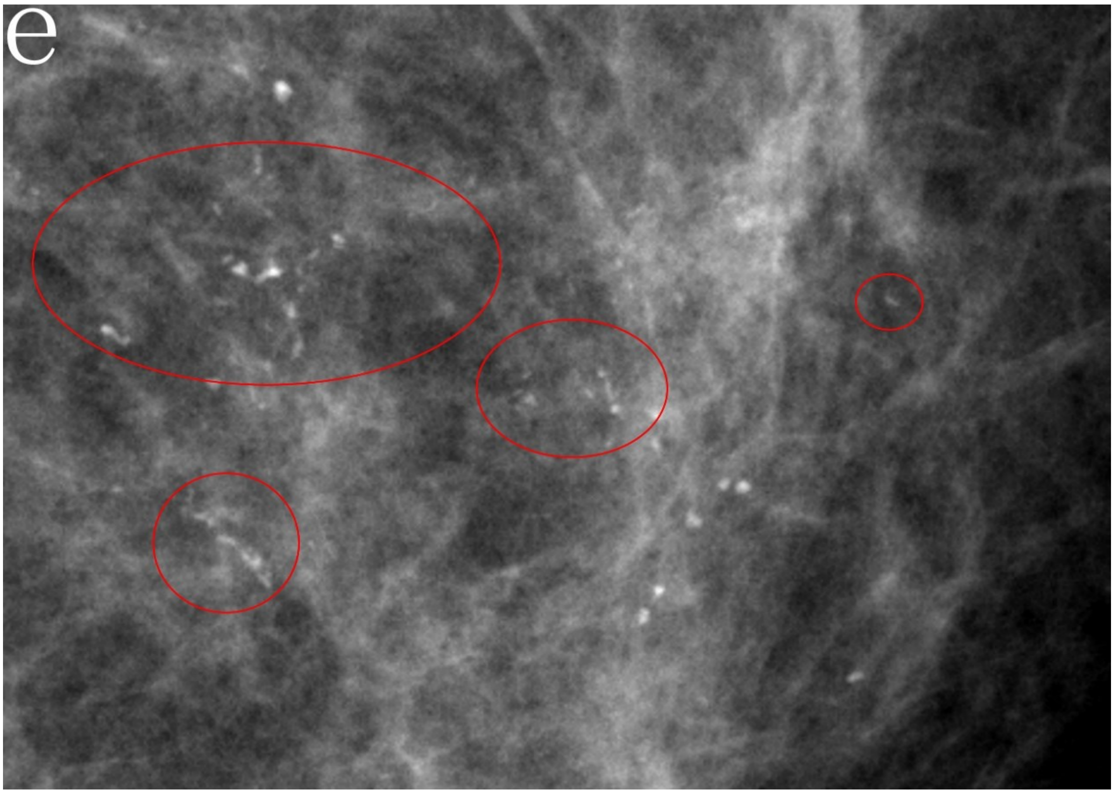

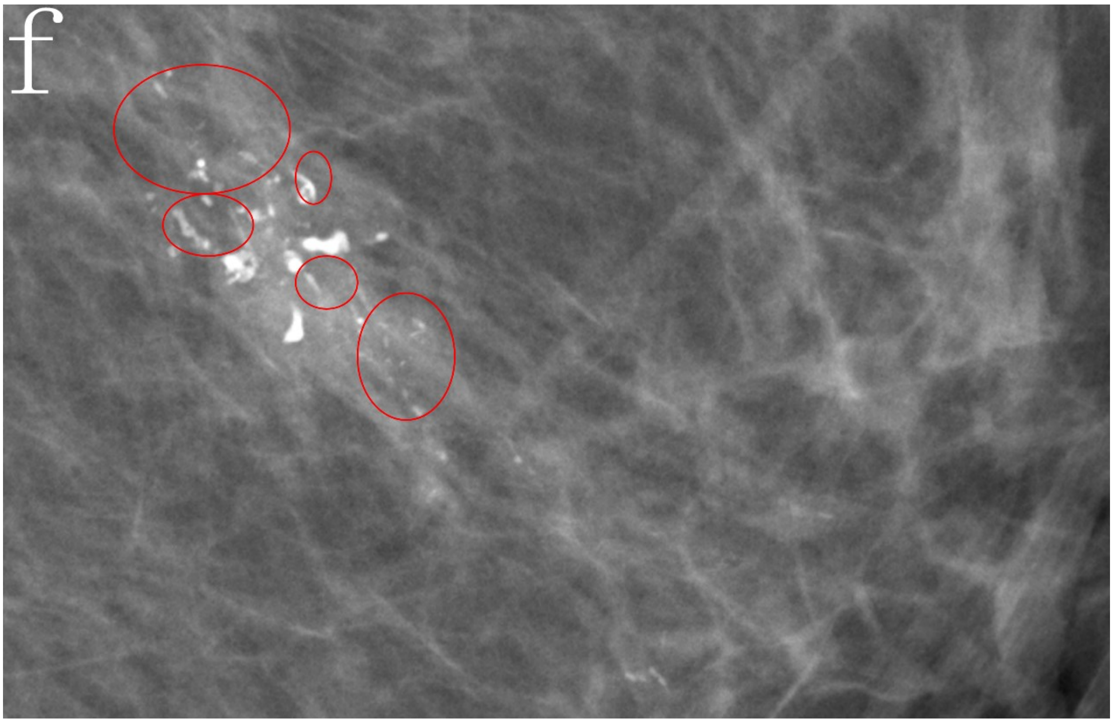

Supplementary FigS1: Six contentious radiographic images were classified after discussion, with the extent of calcification circumscribed (indicated by red circles) as follows: Cases where the calcified area covered less than three-quarters of the total calcification were categorized as the CC-non-predominant group (Figures **a**-**c**); those where calcification extended to at least three-quarters were assigned to the CC-predominant group (Figures **d**-**f**).
